# Association between lipid trajectories during pregnancy and risk of postpartum glucose intolerance after gestational diabetes mellitus: a cohort study

**DOI:** 10.1007/s00592-022-01905-z

**Published:** 2022-07-05

**Authors:** Zhuofan Yang, Zhuyu Li, Yunjiu Cheng, Peisong Chen, Dongyu Wang, Haitian Chen, Wei Chen, Zilian Wang

**Affiliations:** 1 Department of Obstetrics and Gynaecology, The First Affiliated Hospital of Sun Yat-Sen University, 58 Zhongshan Rd II, Guangzhou, 510080 China; 2 Department of Cardiology, The First Affiliated Hospital of Sun Yat-Sen University, Guangzhou, China; 3 Department of Clinical Laboratory, The First Affiliated Hospital of Sun Yat-Sen University, Guangzhou, China

**Keywords:** Lipid trajectory, Glucose intolerance, Gestational diabetes mellitus, Insulin sensitivity

## Abstract

**Aims:**

To assess lipid trajectories throughout pregnancy in relation to early postpartum glucose intolerance in women with gestational diabetes mellitus (GDM).

**Methods:**

This prospective cohort study included 221 Chinese women with GDM who completed plasma lipid test in each trimester of pregnancy and oral glucose tolerance test at 6–9 weeks postdelivery between January 1, 2018 and January 8, 2020. Using the group-based trajectory modeling, total cholesterol (TC), triglyceride (TG), low-density lipoprotein-cholesterol (LDL-c), and high-density lipoprotein-cholesterol(HDL-c) were identified separately as three trajectories: low, moderate, and high trajectory. The associations between lipid trajectories and early postpartum glucose intolerance were all evaluated.

**Results:**

Seventy-three participants developed postpartum glucose intolerance. For patients in low, moderate and high trajectory, the incidence of postpartum glucose intolerance was 38.4%, 34.9%, and 17.9%, respectively. GDM women with lower LDL-c trajectories presented a higher risk of postpartum glucose intolerance. The adjusted odds ratio (95% CI) for glucose intolerance was 3.14 (1.17–8.39) in low LDL-c trajectory and 2.68 (1.05–6.85) in moderate trajectory when compared with the high one. However, TC trajectory was not associated with the risk of postpartum glucose intolerance, nor were TG trajectory and HDL-c trajectory. Moreover, a significant difference of insulin sensitivity was observed in participants with different LDL-c trajectories; participants in high LDL-c trajectory had the highest insulin sensitivity, whereas the women in low LDL-c trajectory had the lowest insulin sensitivity (*P* = 0.02).

**Conclusions:**

The high trajectory of LDL-c during pregnancy may play a protective role on postpartum glucose intolerance in women with GDM. Further studies are warranted to explore the underlying mechanism.

*Trial registration* The study was reviewed and approved by the Institutional Review Board of The First Affiliated Hospital of Sun Yat-sen University (reference number: [2014]No. 93). All participants provided written informed consent forms, and the ethics committee approved this consent procedure.

## Background

Dyslipidemia is a common physiological phenomenon in pregnant women, especially after mid-trimester since increased levels of lipids are essential for fetus development [1]. Nowadays, rising number of researches are exploring the normal range of pregnancy lipids elevation but have not yet achieved consensus [2]. Abnormal maternal lipids have been associated with several adverse consequences such as preeclampsia and large-gestational-age(LGA) infants [3].

Women with GDM refer to those first recognized to suffer glucose intolerance during pregnancy [4], who are at higher risk of both short-term maternal complications and long-term developing metabolic diseases [5–7]. In addition to impaired glucose metabolism, changed levels of lipids were also observed in women with GDM [8]. Current evidence has revealed that hyperlipidemia is much more common in GDM patients. However, related studies mainly focus on one certain trimester and come to various conclusion of how plasma lipid affect glucose metabolism [9–11]. As an important metabolic index, measurement of plasma lipid at single time point is unable to represent the complete metabolic status and reflect the efficacy of lifestyle adjustment of GDM women. What’s more, insufficient information on how lipid profile influences postpartum glucose tolerance was provided by current researches.

This study was performed to investigate the influence of lipid profile trajectories during pregnancy on patients with GDM. To achieve the study goal, we ascertained the longitudinal alterations of lipid profile in GDM women while horizontally compared the associations of different lipid trajectories with postpartum glucose tolerance.

## Methods

### Study subjects

This prospective cohort study was performed as part of an ongoing cohort study in pregnant women who received antenatal care at The First Affiliated Hospital of Sun Yat-sen University. Participants should complete the medical examination annually from January 1, 2018, to January 8, 2020, including three trimesters of pregnancy (9–13, 24–27, and 37–40 gestational weeks) and 6–9 weeks postpartum. Inclusion criteria were GDM women with singleton pregnancy conducted regular pregnancy check-ups and labored in our center and also completed postpartum visit. Exclusion criteria were as follows: (1) pregestational diabetes mellitus (PGDM) including preexisting type 1 or type 2 diabetes mellitus and overt diabetes firstly diagnosed during pregnancy, (2) multiple pregnancy or preterm labor, (3) missing data of lipid profile and glucose tolerance results in any trimester or postpartum visit. Ultimately, a total of 221 participants were recruited in our project, none of whom reported smoking before or during pregnancy. No dyslipidemia or statins treatment before gestation was documented according to medical history provided by all participants.

All participants were referred to the outpatient clinic at our hospital for pregnancy check-ups and consultations. Obstetricians and dietitians would offer professional advice to help adjust lifestyle and achieve targeted glycemic range. Besides assessment during pregnancy, women with GDM were also encouraged to come back for check-ups at 6–9 weeks after delivery.

### Diagnosis of abnormal glucose metabolism

All women without former detected diabetes received a “one-step” 75 g oral glucose tolerance test (OGTT) between 24 and 28 weeks of gestation. The diagnosis of GDM was based on the International Association of Diabetes and Pregnancy Study Groups diagnosis criteria [12].

PGDM includes established diabetes before pregnancy and overt diabetes firstly diagnosed during pregnancy. Established diabetes could be diagnosed easily by self-reported diabetes history or fasting blood glucose ≥ 7.0 mmol/L [4]. Women who underwent 75 g OGTT at 24–28 weeks of gestation with fasting blood glucose(FBG) ≥ 7.0 mmol/L or 2-h value ≥ 11.1 mmol/L were considered as overt diabetes [12].

Postpartum glucose intolerance consists of type 2 diabetes and prediabetes, while the latter one was defined as either impaired fasting glucose or impaired glucose tolerance. To be noticed, the screening strategies are based on the 2020 American Diabetes Association diagnosis criteria of diabetes [4].

## Study assessment

### Blood sample collection

At every regular visit in three trimesters and 6–9 weeks postpartum, blood samples were collected after overnight fasting for 8–10 h and stored at drying vacuum tubes. Before, one and two hours after 75 g glucose load at GDM screening, blood samples were collected. At postdelivery visit, before, half an hour and two hours after 75 g glucose load, blood samples were also collected.

### Assessment of plasma lipid, glucose, and insulin

Each collected sample was measured for TC, TG, LDL-c, and HDL-c levels. OGTT results were assessed by venous blood samples collected at each time point, while insulin levels were measured before half an hour and two hours after 75 g glucose load. HbA1c was measured by high-performance liquid chromatography (VARIANT II; Bio-Rad, Hercules, CA). Serum insulin was measured using chemiluminescence immunoassay (Access®, Beckman Coulter, California, USA). Total cholesterol and triglyceride were assayed by enzymatic colorimetric test. HDL-c and LDL-c were measured using direct enzymatic method. All assays were done in the central laboratory of the First Affiliated Hospital of Sun Yat-sen University.

### Assessment of potential covariates

Baseline characteristics (age, income, prepregnancy weight, height, history of GDM, and family history of diabetes) and obstetrical history of participants were obtained in the first visit of antenatal care by experienced researchers. Prepregnancy BMI was calculated using self-reported prepregnancy weight in kilograms dividing height in meters. Prepregnancy and postpartum overweight were defined based on the World Health Organization definition. The difference between predelivery weight and self-reported prepregnancy weight was gestational weight gain (GWG). HbA1c values were measured before delivery (the day before programmed delivery or on the day of emergency delivery) for further analysis. As ADA has recommended, HbA1c < 6%(42 mmol/mol) is optimal during pregnancy if it can be achieved without significant hypoglycaemia [4]. Thus, HbA1c can be used as a secondary criteria of judging glycemic control effect in pregnancy, which represents the integrated management of blood glucose. In this study, we recognized cases with HbA1c values before delivery ≥ 6.0% (42 mmol/mol) as poorly glycemic controlled ones. As for glucose intolerance valuation, indexes represented insulin sensitivity were also shown in the following report. Homeostasis model assessment-IS (HOMA-IS) was firstly proposed by Turner’s group to reflect insulin sensitivity, while ISOGTT is also used for estimating insulin sensitivity [13]. To be recorded, baseline characters such as intensity of breasting feeding were obtained during the postpartum visit. Postpartum physical activity in patients with GDM was investigated by using the validated International Physical Activity Questionnaires (IPAQ). Types of physical activity include working, transportation, housework, gardening, and leisure activities. Physical activity intensity is divided into walking, moderate activity and high-intensity physical activity. The values of all kinds of physical activity metabolic equivalent (MET) are as follows: high-intensity physical activity = 8.0METs, medium physical activity = 4.0METs, walking = 3.3METs. After assigning the METs to all physical activities, the METs per week(Met-min/W) was evaluated according to the number of days of physical activity (d/w) and the daily accumulated activity time (min/d).

### Statistical analysis

The plasma lipid levels’ progression during pregnancy was analyzed according to GBTM, with “traj” plug-in in Stata 15 [14]. GBTM is designed to divide study subjects into subgroups whose members follow similar change patterns over time of specific parameters of interest, which were TC, TG, LDL-c, and HDL-c in the present study. Using the “traj” program in Stata, we were able to determine how lipid concentrations changed longitudinally. Each case was assigned into one of three subgroups based on the lipid levels throughout pregnancy, which were named Trajectory 1(T1), Trajectory 2(T2), and Trajectory 3(T3), representing low, moderate, and high levels of lipid trajectory, respectively. The shown trajectories were determined by choosing the best-fit number of subgroups as well as the shape of the model. The subgroup number and the shape order eventually identified the best-fit model with highest Bayesian information criterion (BIC), average posterior probability (AvePP) > 0.7, and at least 1% of total cases included in each subgroup.

Baseline characteristics of participants were presented as means and SD for continuous variables and percentages for categorical variables. Tests for differences in means were assessed using unpaired *t*-tests for continuous variables, using χ2 tests for independence for categorical variables. Comparisons of postpartum glucose disturbance among three trajectories of lipid profile during pregnancy were made using multivariable logistic regression analysis adjusted for the following confounders: model I: unadjusted model; model II: adjusted for age, prepregnancy BMI; model III: model II plus adjusted for GWG, insulin treatment during pregnancy, family history of diabetes and HbA1c ≥ 6.0%(42 mmol/mol) before delivery. Unadjusted and adjusted odds ratio (OR) were shown with 95% CI.

To further explore the underlying risk factors that may affect the relationship between blood lipid trajectories and postpartum glucose intolerance, we also performed the subgroup analysis.

All data analyses in this study were conducted using Stata version 15.0(Stata Corp). All *P* values were two-sided and *P*-value of < 0.05 for main effects and interactions was considered statistically significant.

## Results

### Subject baseline characteristics

The baseline characteristics of the cohort study are demonstrated in Table 1. Altogether, two hundred and twenty-one participants with the average age of 33.8 years old as well as prepregnancy BMI of 22.1 kg/m^2^ were included in our study, among which 85 participants (38.5%) were of advanced age and 73 women (34%) were defined as overweight. The mean OGTT results were 4.5, 9.8, and 8.9 mmol/L for fasting, 1 h and 2 h after glucose load, respectively. Furthermore, the mean HbA1c value before delivery is 5.3% (34 mmol/mol), with 14 participants (6.3%) defined as poorly glycemic-controlled cases.

**Table 1:** Characteristics throughout pregnancy among 221 women with GDM in different LDL-c trajectories

Variables^*^	All (*n* = 221)	Trajectory 1^†^ (*n* = 73)	Trajectory 2^†^ (*n* = 109)	Trajectory 3^†^ (*n* = 39)	*P* value^§^
Age(y)	33.8 ± 4.3	33.5 ± 4.2	33.5 ± 4.3	35.1 ± 4.7	0.11
Advantaged maternal age(%)	85(38.5)	26(35.6)	42(38.5)	17(43.6)	0.71
Income					0.68
Low	22(10.0)	10(13.7)	9(8.3)	3(7.7)	
Median	75(34.0)	23(31.5)	40(36.7)	12(30.8)	
High	124(56.0)	40(54.8)	60(55.0)	24(61.5)	
Prepregnancy BMI (kg/m^2^)	22.1 ± 3.1	22.6 ± 3.5	21.8 ± 2.9	21.9 ± 3.0	0.22
Prepregnancy overweight (%)	73(33.0)	31(42.5)	27(24.8)	15(38.5)	0.03
Multiparity	120(54.3)	38(52.1)	56(51.4)	26(66.7)	0.23
History of GDM	39(32.5)	16(42.1)	18(32.1)	5(19.2)	0.16
Family history of diabetes	78(35.3)	31(42.5)	38(34.9)	9(23.1)	0.12
Gestational weight gain(kg)	10.9 ± 4.0	11 ± 4.0	10.8 ± 4.1	11 ± 3.9	0.96
Insulin treatment during pregnancy	6(2.7)	3(4.1)	3(2.8)	0(0)	0.44
Hypertensive disorders during pregnancy	12(5.4)	3(4.1)	8(7.3)	1(2.6)	0.44
Gestational age at delivery(weeks)	38.7 ± 1.1	38.8 ± 1.0	38.6 ± 1.3	38.8 ± 1.1	0.36
Cesarean delivery	128(57.9)	41(56.2)	63(57.8)	24(61.5)	0.86
Neonatal gender(male)	125(56.6)	40(54.8)	67(61.5)	18(46.2)	0.24
Neonatal birth weight	3121.6 ± 408.6	3069.3 ± 410.1	3128.3 ± 413.3	3200.8 ± 388.3	0.26
LDL-c in first trimester(mmol/L)	2.9 ± 0.6	2.4 ± 0.4	3.0 ± 0.4	3.5 ± 0.5	< 0.01
LDL-c in second trimester(mmol/L)	3.5 ± 0.8	2.7 ± 0.4	3.6 ± 0.4	4.6 ± 0.5	< 0.01
LDL-c in third trimester(mmol/L)	3.7 ± 0.8	2.8 ± 0.4	3.8 ± 0.4	4.8 ± 0.5	< 0.01
OGTT during pregnancy(mmol/L)					
FPG	4.5 ± 0.5	4.6 ± 0.5	4.5 ± 0.5	4.5 ± 0.5	0.86
Glycemia 1 h	9.8 ± 1.4	9.8 ± 1.4	9.8 ± 1.5	10.0 ± 1.2	0.77
Glycemia 2 h	8.9 ± 1.3	8.9 ± 1.5	8.9 ± 1.3	8.9 ± 1.1	0.96
HbA1c value before delivery(%)	5.3 ± 0.4	5.3 ± 0.5	5.4 ± 0.4	5.4 ± 0.3	0.12
The frequency of HbA1c ≥ 6.0%(42 mmol/L) before delivery	14(6.3)	5(6.8)	7(6.4)	2(5.1)	0.94

^*^Continuous variables were presented as mean (SD)

^†^Trajectories 1, 2, 3 refer to LDL-c trajectory

^§^*P* values were calculated by Kruskal–Wallis test for continuous variables and Chi-square test for categorical variables

**Fig. 1:**
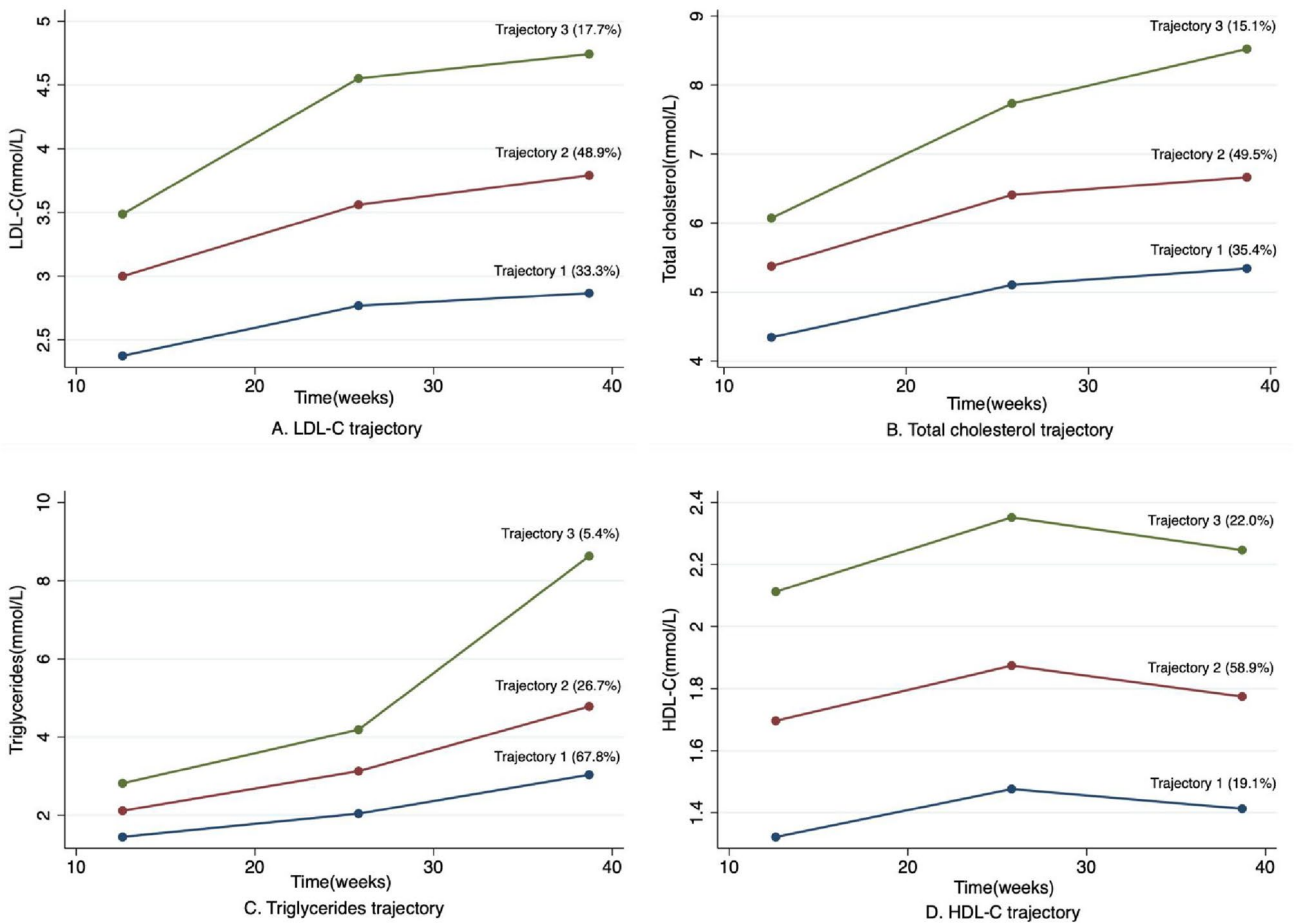
Best-fit lipid trajectories during pregnancy

Figure 1 shows the three trajectories of each lipid (TC, TG, LDL-c, and HDL-c) during pregnancy established as low, moderate, and high trajectory using GBTM. Throughout the pregnancy, all three trajectories showed elevation except for HDL-c, which firstly raised to reach the maximum at mid-trimester and slightly fell off at late-pregnancy. Among four sets of trajectories, the high-level trajectory of TC, TG, and LDL-c included the least number of individuals, while the low-level trajectory of HDL-c showed to have the least members, which indicated that most participants only suffered from relatively slight dyslipidemia during pregnancy.

### Subjects’ characteristics at postpartum period

The postpartum data of study subjects are depicted in Table 2. The postpartum BMI was slightly increased to 22.6 kg/m^2^ with average weight retention of 1.4 kg among all participants. The number of overweight subjects was 86, which remained the same compared with prepregnancy data. After conducting questionnaire survey during postpartum follow-ups, no significant difference between physical activity after delivery and LDL-c trajectories was detected. The postpartum OGTT was performed at 7.7 ± 1.6 weeks after delivery. According to OGTT results after delivery, 73 participants developed glucose intolerance postpartum, which means almost a third of GDM women developed glucose intolerance in our study. The mean incidence rates of IFG, IGT, prediabetes, type 2 diabetes among women with postpartum OGTT results were 0.9%, 29.9%, 30.8%, and 2.3%, respectively. Instead of falling back to starting levels, the LDL-c levels postpartum in three trajectories were all found increased markedly with the rise of LDL-c during pregnancy (*P* < 0.01), which were highest in high trajectory and lowest in low trajectory.

**Table 2:** Characteristics after delivery among 221 women with GDM in different LDL-c trajectories

Variables	All (*n* = 221)	Trajectory 1 (*n* = 73)	Trajectory 2 (*n* = 109)	Trajectory 3 (*n* = 39)	*P* value
Postpartum BMI(kg/m^2^)	22.6 ± 3.1	23.2 ± 3.6	22.3 ± 2.7	22.4 ± 3.3	0.13
Postpartum overweight (%)	86(38.9)	35(47.9)	36(33.0)	15(38.5)	0.19
Postpartum waist circumference(cm)	85.2 ± 7.2	85.9 ± 8.3	85.0 ± 6.4	84.6 ± 7.0	0.6
Postpartum waist/hip ratio	0.91 ± 0.05	0.91 ± 0.06	0.91 ± 0.05	0.90 ± 0.04	0.32
Postpartum weight retention(kg)	1.4 ± 3.5	1.7 ± 3.8	1.3 ± 3.4	1.2 ± 3.3	0.75
Postpartum weight retention (%)	140(63.3)	49(67.1)	65(59.6)	26(66.7)	0.74
Postpartum physical activity (Mets)	546.75(210,985.5)	688(287.3,1340)	500.8(202.1,962.3)	264(107.8,789)	0.73
Intensity of breastfeeding					0.18
Mostly exclusive breastfeeding	79(35.7)	24(32.9)	46(42.2)	9(23.1)	
Half breastfeeding and half formula feeding	119(53.8)	40(54.8)	52(47.7)	27(69.2)	
Mostly formula breastfeeding	23(10.4)	9(12.3)	11(10.1)	3(7.7)	
Postpartum LDL-c(mmol/L)	3.5 ± 0.8	3.0 ± 0.5	3.6 ± 0.6	4.2 ± 0.8	< 0.01
Postpartum OGTT(mmol/L)					
FPG	4.7 ± 0.6	4.7 ± 0.6	4.6 ± 0.5	4.6 ± 0.5	0.23
Glycemia 2 h	7.1 ± 1.8	7.2 ± 2.0	7.2 ± 1.8	6.6 ± 1.3	0.17
IFG	2(0.9)	0(0)	2(1.8)	0(0)	0.36
IGT	66(29.9)	26(35.6)	33(30.3)	7(17.9)	0.15
Prediabetes	68(30.8)	26(35.6)	35(32.1)	7(17.9)	0.14
Type 2 diabetes	5(2.3)	2(2.7)	3(2.8)	0(0)	0.58
Postpartum glucose intolerance	73(33.0)	28(38.4)	38(34.9)	7(17.9)	0.08
HOMA-S	1.3 ± 0.9	1.0 ± 0.5	1.3 ± 0.9	1.5 ± 1.1	0.02
ISOGTT	26.1 ± 15.2	22.5 ± 11.3	27.6 ± 16.7	28.6 ± 16.2	0.04
IGI/HOMA-IR	11.7 ± 10.0	12.3 ± 13.0	10.6 ± 8.1	11.7 ± 10.0	0.23
HOMA-IR	1.2 ± 1.1	1.3 ± 1.0	1.2 ± 1.2	1.0 ± 0.7	0.28

### Association of lipid trajectories with postpartum dyslipidemia

As shown in Table 3, when compared with T1, all lipids except HDL-c presented elevated the incidence of postdelivery dyslipidemia with significant differences. Risk of developing dyslipidemia after giving birth became significantly higher in GDM women with a higher trajectory of lipid profile such as TC, TG, and LDL-c. After controlling potential confounders, the above significance still existed. No difference was established between different trajectories of HDL-c with risk of postdelivery dyslipidemia in the study.

**Table 3:** Associations of lipid profile trajectories with postpartum dyslipidemia

	Postpartum dyslipidemia	Model I^*^ OR(95%CI)	*P* value	Model II^†^ OR(95%CI)	*P* value	Model III^‡^ OR(95%CI)	*P* value
LDL-c during pregnancy	Trajectory 1	Reference		Reference		Reference	
	Trajectory 2	3.71(1.80–7.67)	< 0.01	4.10(1.94–8.67)	< 0.01	4.30(2.01–9.16)	< 0.01
	Trajectory 3	11.4(4.56–28.7)	< 0.01	11.9(4.63–30.8)	< 0.01	13.5(5.08–35.7)	< 0.01
TC during pregnancy	Trajectory 1	Reference		Reference		Reference	
	Trajectory 2	2.20(1.15–4.21)	0.02	2.19(1.13–4.25)	0.02	2.33(1.18–4.58)	0.02
	Trajectory 3	7.21(2.89–18.01)	< 0.01	7.17(2.83–18.2)	< 0.01	7.74(2.99–20.0)	< 0.01
TG during pregnancy	Trajectory 1	Reference		Reference		Reference	
	Trajectory 2	2.48(1.33–4.63)	0.01	2.32(1.23–4.37)	0.01	2.30(1.21–4.40)	0.01
	Trajectory 3	4.47(1.28–15.6)	0.02	4.20(1.19–14.8)	0.03	4.12(1.15–14.7)	0.03
HDL-c during pregnancy	Trajectory 1	Reference		Reference		Reference	
	Trajectory 2	0.81(0.38–1.73)	0.58	0.84(0.38–1.82)	0.65	0.79(0.35–1.78)	0.57
	Trajectory 3	0.84(0.38–1.82)	0.65	1.51(0.59–3.79)	0.39	1.44(0.56–3.68)	0.5

^*^Model I: Model without adjustment

^†^Model II: Adjusted for age and prepregnancy BMI

^‡^Model III: Adjusted for variables in model II plus GWG, insulin treatment during pregnancy, FPG on pregnancy OGTT, Glycemia 2 h on pregnancy OGTT, family history of diabetes, and HbA1c ≥ 6.0% before delivery

### Association of lipid trajectories with postpartum glucose intolerance

Among all these data represented glucose metabolism of GDM patients after giving birth, we noticed that the rate of glucose intolerance slightly decreased with ascending trajectory of LDL-c(*P* = 0.08). The incidences of glucose intolerance decreased along with the low, moderate, and high levels of LDL-c trajectories, which were 38.4%, 34.9%, and 17.9%, respectively. It was evident the incidence of glucose intolerance in low trajectory (38.4%) was significantly higher compared with that in high trajectory (17.9%) (*P* = 0.03). Accordingly, HOMA-IS, the index represented insulin sensitivity, increased significantly across the three subgroups from low to high trajectory (*P* = 0.02). Similarly, the postpartum level of ISOGTT was highest in the low trajectory and lowest in the high trajectory (*P* = 0.04).

As for other lipids including TC, TG, and HDL-c, no different risks of developing glucose intolerance after delivery were found between various levels of trajectories (Table 4).

**Table 4:** Associations of lipid profile trajectories during pregnancy with postpartum glucose intolerance

	Postpartum glucose intolerance	Model I OR(95%CI)	*P* value	Model II OR(95%CI)	*P* value	Model III OR(95%CI)	*P* value
LDL-c during pregnancy	Trajectory 1	2.84(1.11–7.31)	0.03	3.30(1.25–8.72)	0.02	3.14(1.17–8.39)	0.02
	Trajectory 2	2.45(0.99–6.06)	0.05	2.87(1.13–7.29)	0.03	2.68(1.05–6.85)	0.04
	Trajectory 3	Reference		Reference		Reference	
TC during pregnancy	Trajectory 1	1.46(0.59–3.59)	0.41	1.60(0.64–4.02)	0.31	1.47(0.57–3.74)	0.42
	Trajectory 2	1.21(0.51–2.88)	0.67	1.26(0.52–3.04)	0.6	1.24(0.51–3.01)	0.64
	Trajectory 3	Reference		Reference		Reference	
TG during pregnancy	Trajectory 1	1.07(0.31–3.72)	0.91	1.18(0.34–4.17)	0.79	1.35(0.38–4.88)	0.64
	Trajectory 2	0.78(0.21–2.96)	0.72	0.77(0.20–2.95)	0.7	0.81(0.20–3.21)	0.76
	Trajectory 3	Reference		Reference		Reference	
HDL-c during pregnancy	Trajectory 1	1.88(0.72–4.93)	0.12	1.86(0.69–5.00)	0.22	1.58(0.57–4.41)	0.38
	Trajectory 2	1.71(0.80–3.67)	0.17	1.71(0.79–3.71)	0.17	1.76(0.80–3.87)	0.16
	Trajectory 3	Reference		Reference		Reference	

For baseline characters among LDL-c trajectories, a lower prepregnancy overweight rate was observed in moderate trajectory (24.8%), while higher ones were found in low (42.5%) and high trajectories (42.5%), which were statistically different (*P* = 0.03). Other potential risk factors both during gestation (Table 1) and after delivery (Table 2) were found balanced between three trajectories. Since medication for treating hyperlipidemia is not available for pregnant women in China, none of our participants acquired lipid concentration reduction with medication.

Table 4 shows the unadjusted and adjusted OR of postpartum glucose intolerance with the high trajectory as the reference in multivariable logistic regression models. The risks of postpartum aberrant glucose tolerance were increased markedly in low trajectory and moderate trajectory, in which the unadjusted odds ratio was 2.84 (95% CI: 1.11–7.31) and 2.45 (95% CI: 0.99–6.06), respectively (Table 4, Model I). The increased tendency still held after adjusting confounders (Table 4, Model II, Model II). In Model II, significantly elevated incidences of postpartum glucose intolerance were revealed in low trajectory (OR, 3.30; 95% CI, 1.25–8.72) and moderate trajectory (OR, 2.87; 95% CI, 1.13–7.29). Moreover, compared with the reference, the OR of low trajectory was 3.13 (95% CI: 1.17–8.39) and of moderate trajectory was 2.68(95% CI: 1.05–6.85) with statistically significance (*P* = 0.02 and 0.04, respectively) after adjusting for underlying confounders including OGTT.

**Fig. 2:**
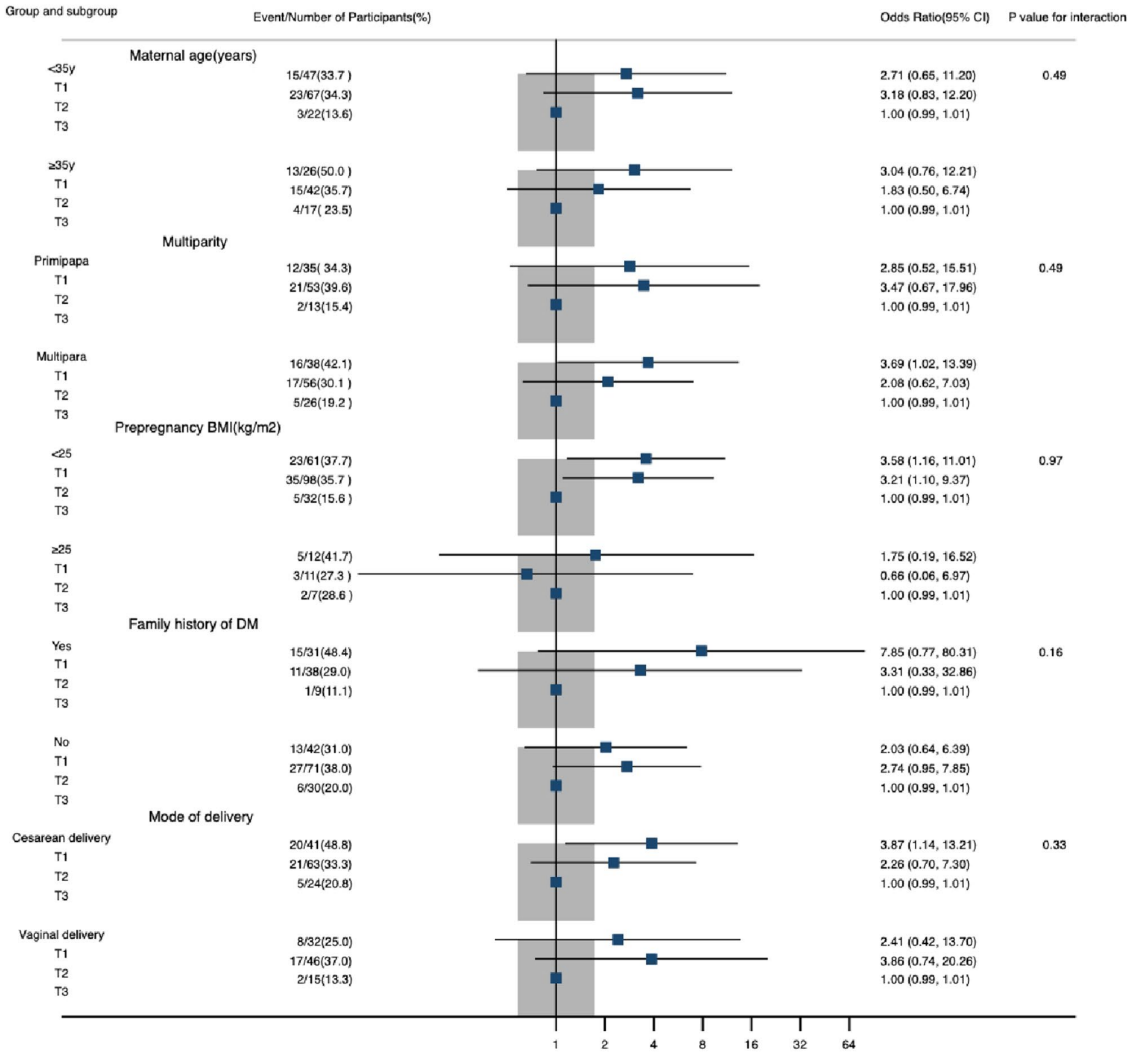
Association between LDL-c trajectory and postpartum glucose intolerance in subgroups. Models adjusted for maternal age, prepregnancy BMI, family history of DM, GWG, HbA1c ≥ 6.0% before delivery, insulin treatment during pregnancy (subgroup used in stratification is not included in the model)

At last, Fig. 2 shows the association between LDL-c trajectory and maternal glucose intolerance by stratified analysis. No significant interaction effects were identified for potential risk factors including maternal age, multiparity, prepregnancy BMI, family history of diabetes, and mode of delivery.

## Discussion

In the present observational cohort study, we revealed that women with GDM in the low trajectory of LDL-c during pregnancy were 3.14 times more likely to suffer glucose intolerance postpartum compared with GDM women in the high LDL-c trajectory. LDL-c trajectory during pregnancy exhibited an inverse correlation with the risk of postpartum glucose intolerance, which seemed to be counterintuitive, while different trends of TC, TG and HDL-c displayed insignificant differences in developing postpartum glucose intolerance.

Nowadays, the relationship between altered lipid concentrations during gestation and the risk of metabolic diseases is now receiving widespread coverage [15]. Evidence has indicated that lipid profile such as TG, HDL-c, and TG/HDL-c ratio plays predictive roles in GDM [16]. Besides metabolic dysfunction during pregnancy, evidence is growing that dyslipidemia of GDM women may influence the future risk of metabolic disorders [17]. Toescu V's work revealed that hyperlipidemia may be related to long-term cardiovascular diseases, considering elevated plasma lipid level contributes to increased oxidative stress and injury [18]. Abnormal LDL-c and TG after delivery were considered as contributors for developing insulin resistance in women with GDM [19]. However, limited studies have payed attention on pregnancy dyslipidemia and postdelivery glucose tolerance, which makes their relationship unrevealed. Actually, the studies discussed relationship between gestational lipid and postpartum glucose metabolism were limited and inconsistent. According to a recent cohort study based on Chinese population, GDM patients with high TG tertile during the second trimester were associated with a significantly increased risk of postpartum glycometabolism [20]. Another related study carried out by Pei Xiaocao and his team also found that GDM patients with abnormal postpartum glycometabolism were more likely to have higher TG and LDL-c in the second trimester [21]. However, GDM patients who located at low cholesterol quartiles at the time of GDM diagnosis were reported to have higher risk of abnormal glucose metabolism after delivery [22]. All of the above results indicated that the relationship between pregnancy lipid and postpartum glucose metabolism was yet to be fully revealed.

In our cohort, the results suggested a negative relationship between LDL-c trajectory and risk of developing glucose intolerance, while denied the significant association between TC, TG as well as HDL-c and postpartum glucose intolerance.

These inconsistent and astonishing results may impute confounders and a relative small sample size. However, researchers have found that LDL-c reduction with statin therapy resulted in a modest increase of risk of new-onset diabetes mellitus (NODM) [23], which provided our unusual findings with possible theoretical support and scientific hypothesis. Increasing transportation of LDL-c into the liver, pancreas, and other tissue through low-density protein receptor (LDLR) was one of the mechanisms of how statin reduced LDL-c level in plasma. This could lead to excessive LDL-c storage in the pancreas and cause ß‐cell dysfunction and subsequently impaired insulin production [24]. Indeed, the affinity of LDLR is influenced by small and dense LDL-c particles and the oxidation they induced, which means small and dense LDL-c may promote LDL-c removal into the pancreas [25]. Evidence showed that the rising level of blood LDL-c, especially in hyperglycemia women, can inhibit LDLR mediated blood cholesterol clearance by affecting LDLR expression [26]. Invoking LDL-c as an inhibitor of pancreatic cholesterol accumulation by down-regulating LDLR may help explain why high level of LDL protect GDM women from damaged glucose tolerance.

Based on GBTM-analyzed lipid profile trajectories, our study showed LDL-c trajectory during pregnancy was inversely associated with postpartum glucose intolerance in women with GDM. Besides, among different trajectories of other lipid profiles such as TC, TG, and HDL-c, no significant difference in postdelivery glucose tolerance was revealed. All in all, present results revealed the possibility that LDL-c may play a role in restoring glucose tolerance in GDM women. Although the above discussion listed some potential mechanisms, the mechanistic basis is not elucidated yet. Thus, to bring the underlying mechanisms to light, basic research should be carried out in the further.

The strengths of our study included a longitudinal design covering the entire pregnancy and also postpartum, using GBTM to analyze the underlying tendency of longitudinal data. To the best of our knowledge, this is the first study exploring the relationship between pregnancy lipid profile and postpartum glucose tolerance based on longitudinal lipid profile change patterns. Nevertheless, this study has its limitations. Firstly, the study advanced several possible mechanisms without experiment data to truly support them. What’s more, the relatively small sample size and limited race of this study will require further analyses with a large sample size and a wide range of ethnic groups. Last but not least, data considering maternal exercise before and during gestation have not been collected in the present study. Actually, preconception and pregnancy physical exercise can ameliorate the deleterious effects of maternal high-fat diet [27] as well as reduce the risk of developing glucose intolerance [28], which indicates the importance of including and analyzing relevant data in our further study.

## Conclusion

Our study demonstrated three LDL-c trajectories (low, moderate, and high) during pregnancy and reported that the lower LDL-c trajectory surprisingly contributes to a higher risk of developing impaired glucose intolerance. The results suggest that the longitudinal trajectory of LDL-c has an impact on postpartum glucose metabolism, especially insulin sensitivity of women with GDM. Further studies designed to investigate the underlying mechanism of how LDL-c negatively influenced postpartum glucose tolerance will help provide valuable insights for clinical intervention of gestational hyperlipidemia.

## Data Availability

Some or all data generated or analyzed during this study are included in this published article or in the data repositories listed in References.

## Abbreviations

GDM: Gestational diabetes mellitus
GBTM: Group-based trajectory modeling
TC: Total cholesterol
TG: Triglyceride
LDL-c: Low-density lipoprotein-cholesterol
HDL-c: High-density lipoprotein-cholesterol
PGDM: Pregestational diabetes mellitus
OGTT: Oral glucose tolerance test
FBG: Fasting blood glucose
HOMA-IS: Homeostasis model assessment-IS
BIC: Bayesian information criterion
AvePP: Average posterior probability
NODM: New-onset diabetes mellitus
LDLR: Low-density protein receptor
